# Supplementary material for: Comparing posttraumatic growth in mothers after stillbirth or early miscarriage
Source: PLoS One. 2022 Aug 8;17(8):e0271314. doi: 10.1371/journal.pone.0271314 (PMC9359608; doi:10.1371/journal.pone.0271314)
Supplement: Attachment — Submitted filename: Response to reviewers.docx [file pone.0271314.s001.docx]

Avon & Wiltshire Mental Health Partnership NHS Trust

c/o Kingsdown Fort

Bristol

BS6 5UB

Email: sarah.stacey1@nhs.net

PLOS ONE

17^th^ May 2022

Dear Editor and reviewers,

Many thanks for your review of our manuscript, and thoughtful comments and feedback. Please see copied below your comments and our responses.

Kind regards,

Sarah Stacey (on behalf of all co-authors)

**Reviewers' comments:**

**Reviewer #1:**

**This well written article compares posttraumatic growth in women after stillbirth or early miscarriage in 120 women. The manuscript is logic and easy to follow and comes to sound conclusions. It addresses a topic where one still needs more knowledge.**

**A few uncertainties need attention:**

Many thanks for highlighting these, our comments are below.

**1. Table 1. Line 261 refers to ‘being currently pregnant’ but it is not reflected in the table.**

Response: Many thanks for highlighting this. We have now included the data for the number of participants in each group who were pregnant at the time of the study into Table 1.

**2. Table 2. The legend refers to ‘GAD-7’ and ‘PHQ’ but it is not in the table.**

Response: Many thanks for spotting this. We have deleted the reference to these questionnaires from the legend.

**3. The words in brackets following ’Model of Growth in Grief’ differ. Please compare line 157 with line 141. The meaning of ‘CAB’ is unclear.**

Response: With thanks again. We have removed the acronym and amended line 157 to be the same as line 141. Both lines now reflect that the current study particularly explored experiences of challenge to assumptive beliefs (CAB), rumination and disclosure in relation to early miscarriage and stillbirth.

**Reviewer #2:**

**Thanks for this submission.
In my hospital, all women with SB could benefit from a psychiatrist consultation. I guess it is a way to incresase the rate of PTG. In the other hand, women with early miscarriage don't benefit from that.**

**Did you explore psychiatrist intervention in your population?**

Many thanks for your insightful question. We did not explore the possibility of impact of psychiatrist intervention in the current study, although agree that this could be worthy of further investigation. This study was conducted online so participants will have been treated at many different hospitals and, therefore, will likely have received differing kinds of care after their loss.

Psychiatrist intervention could be considered for any hospital patient who was thought to be experiencing severe mental health difficulties and could be accessed through the hospital-based Liaison Psychiatry service which exists in most larger acute hospitals in England. We did not gather data as to whether any of the participants in this study were referred to this kind of service at the time of their loss.
